# Strategies to Mitigate Variability in Engineering Human Nasal Cartilage

**DOI:** 10.1038/s41598-017-06666-2

**Published:** 2017-07-26

**Authors:** Stephen H. J. Andrews, Melanie Kunze, Aillette Mulet-Sierra, Lynn Williams, Khalid Ansari, Martin Osswald, Adetola B. Adesida

**Affiliations:** 1 Laboratory of Stem Cell Biology and Orthopaedic Tissue Engineering, Divisions of Orthopaedic Surgery and Surgical Research, Department of Surgery, University of Alberta, Li Ka Shing Centre for Health Research Innovation, Edmonton, AB Canada; 2 Division of Otolaryngology-Head and Neck Surgery, Department of Surgery, University of Alberta Hospital, Edmonton, AB Canada; 3 Institute for Reconstructive Sciences in Medicine (iRSM), Misericordia Community Hospital, Edmonton, AB Canada

**Keywords:** Cell growth, Mesenchymal stem cells

## Abstract

Skin cancer and its associated treitments can have devastating consequences for survivors; this is particularly true when cancer occurs on the nose. Recent work has applied cell-based tissue engineering (TE) strategies to develop nasal cartilage constructs for reconstruction of the nose. In this study, we have generated human nasal cartilage on a clinically approved collagen scaffold to investigate the donor-to-donor variability of TE cartilage and evaluated strategies to mitigate it. We also evaluated the gene expression of the family of fibroblast growth factor receptors (FGFR1-4) and their association with tissue quality. *FGFR1* was significantly positively correlated with GAG/DNA; a measure of chondrogenic capacity. We implemented two strategies: hypoxic culture and co-culture with mesenchymal stromal cells (MSCs) to increase tissue quality. Total glycosaminoglycan (GAG) content varied significantly between donors initially, with >10–fold difference between the best and worst donor tissue. Our co-culture strategy was able to increase TE construct quality from poor quality donor tissue while supressing hypertrophy relative to MSCs alone. However, no differences were observed with the use of hypoxic culture. Tissues generated using co-culture with MSCs became vascularized and calcified *in vivo*, demonstrating a non-stable cartilage phenotype in co-culture and MSCs cartilage constructs.

## Introduction

More than 3 million people were treated for skin cancers in 2015 in the United States alone^1^. While the vast majority of these cancers are treatable, the effects of this disease and its attending treatments can have devastating morbidity for survivors. A large percentage, approximately 36% of all skin-cancer cases, occur on a patient’s nose^2^. When cancer is diagnosed on the face, and in particular on the nose, treatment options often lead to loss of function and disfigurement^3^. The nose is the most prominent and conspicuous feature of the face, making deformities difficult to conceal, which can lead to emotional distress along with the concominant loss of nasal breathing function.

**Figure 1:**
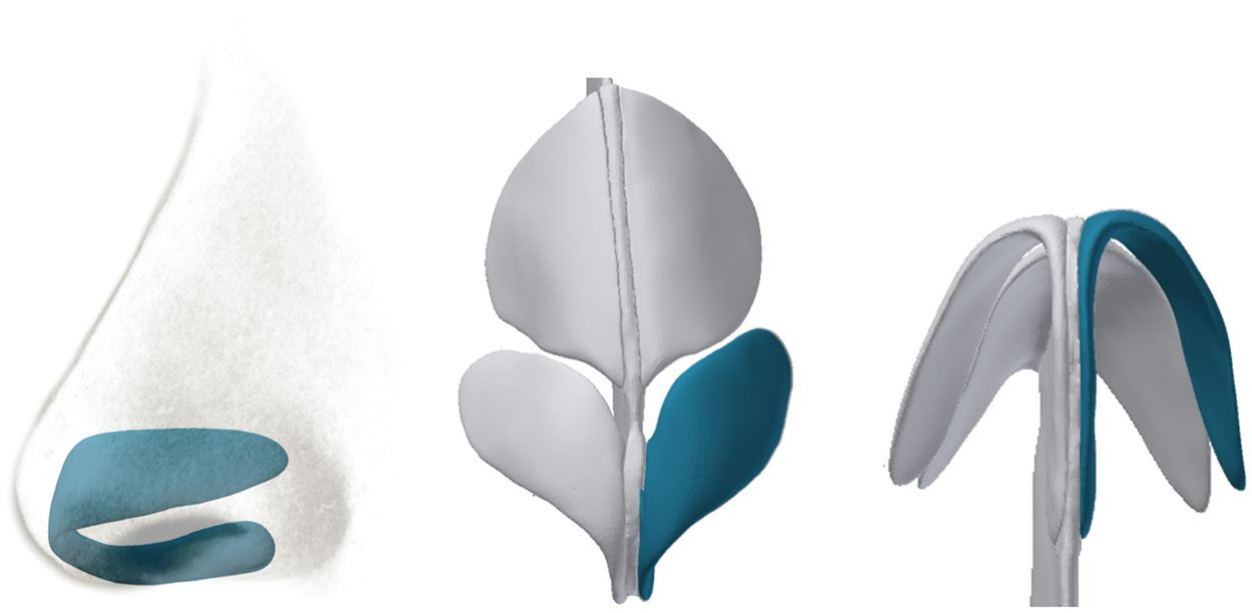
Illustration of the cartilaginous structure of the nose, with specific focus on the alar cartilages (also known as Lower Later Cartilages), shown in blue. Left: lateral view, with illustration of the cartilage position within the nose. Centre: Anterior view of the cartilaginous structure of the nose captured using computed tomography. Right: Inferior view demonstrating the curvature of the alar cartilages.

The greater alar cartilages, as the name suggests, are paired wing shaped structures, that form the outer eminence of the nostril (whose confluence imparts a form similar to McDonald’s arches) (Fig. 1). These structures, also known as the lower lateral cartilages (LLC), provide the dual function of conferring the intricate nuances of the aesthetic shape of the nasal tip and the nostrils while providing critical support to the nasal vestibular airway^4^. The nasal septum is primary structural cartilage in the centre of the nose and is of paramount importance, as it is primarily responsible for setting the dorsal and caudal projection of the external nose in 3-dimensional space. When these cartilages are sacrificed during the treatment of a nasal tip skin cancer with surgery or radiation or both, it can be extremely challenging to replicate or even emulate this complex cartilaginous structure with standard reconstructive techniques^2, 5^. Currently, reconstruction of the LLCs or nasal septum requires the harvest of free cartilage grafts from either the concha of the ear or the costal cartilage of the ribs^2, 6^. The procedures themselves can impart significant donor site morbidity for the patient such as surgical site pain, infection, scarring, deformity, and even life threatening complications such as pneumothorax when harvesting rib cartilage^7^. Once the cartilage graft has been obtained, it must be carved to the appropriate shape by the surgeon in the operating suite which can be extremely time consuming and technically challenging, increasing the cost of the overall reconstruction. Despite meticulous carving, with time, such *in situ* grafts may still undergo reabsorption, warping (bending with time due to instrinsic forces within the native cartilage)^8^, and shifting in position due to scar contracture, leading to less than ideal aesthetic and functional results for the nose.

Tissue engineering (TE) is a promising strategy to replace the need for harvesting cartilage from other anatomical sites and eliminating secondary donor morbidity for the patient. Nasal chondrocytes (NC) have been a target for engineering both nasal cartilage and articular cartilage^9–12^. Nasal chondrocytes can be easily isolated and harvested from a biopsy of the nasal septum. These cells can then be expanded in *in vitro* culture with growth factors that enhance their capacity to redifferentiate and express the functional extracellular matrix of native nasal cartilage^11^. Primary nasal chondrocytes have been shown to still produce cartilaginous matrix after two cycles or passages of expansion in cell culture^13^. Furthermore, nasal chondrocytes isolated from nasal septum have been used to engineer alar cartilage replacements in a first of its kind human trial, involving 5 patients with non-melanoma skin cancer on the alar lobule^11^. While successful in its clinical outcomes, this trial did demonstrate significant variability between donors in the quality of the tissue and the amount of glycosaminoglycan (GAG) produced *in vitro*.

Primary nasal chondrocytes when co-cultured with bone marrow derived mesenchymal stromal cells (BM-MSCs) have been shown to increase GAG production, collagen type II and decrease collagen X in pellet culture models^12^. However, cartilage tissue generated using BM-MSCs have been shown to undergo premature hypertrophy and calcification in ectopic implantation in animal models^14^. Culture in low oxygen conditions has also been demonstrated to increase the redifferentiation and chondrogenic capacity of expanded nasal chondrocytes in pellet models^15^. Therefore, the purposes of this study were three-fold. First, we aimed to characterize and evaluate the variability amongst donors, in the quality of tissue generated from expanded nasal chondrocytes on a commercially available, clinically approved porous collagen scaffold (Chondro-Gide) (**Objective 1**). The quality of the engineered tissue constructs was evaluated through histological, immunofluorescence and molecular analyses. The tissue engineerd constructs were assessed against normal nasal septal cartilage as a benchmark for comparison. Second, we aimed to reduce inter-subject variability using hypoxic culture in 3% O_2_, as well as using co-culture with BM-MSCs (**Objective 2**). Third, to determine the clinical translatability of our culture techniques using BM-MSC and hypoxia, we evaluated the phenotypic stability of our cartilage constructs through subcutaneous implantation in a nude mouse model (**Objective 3**).

## Results

### Objective 1: Examination of Inter-subject Variability

**Figure 2:**
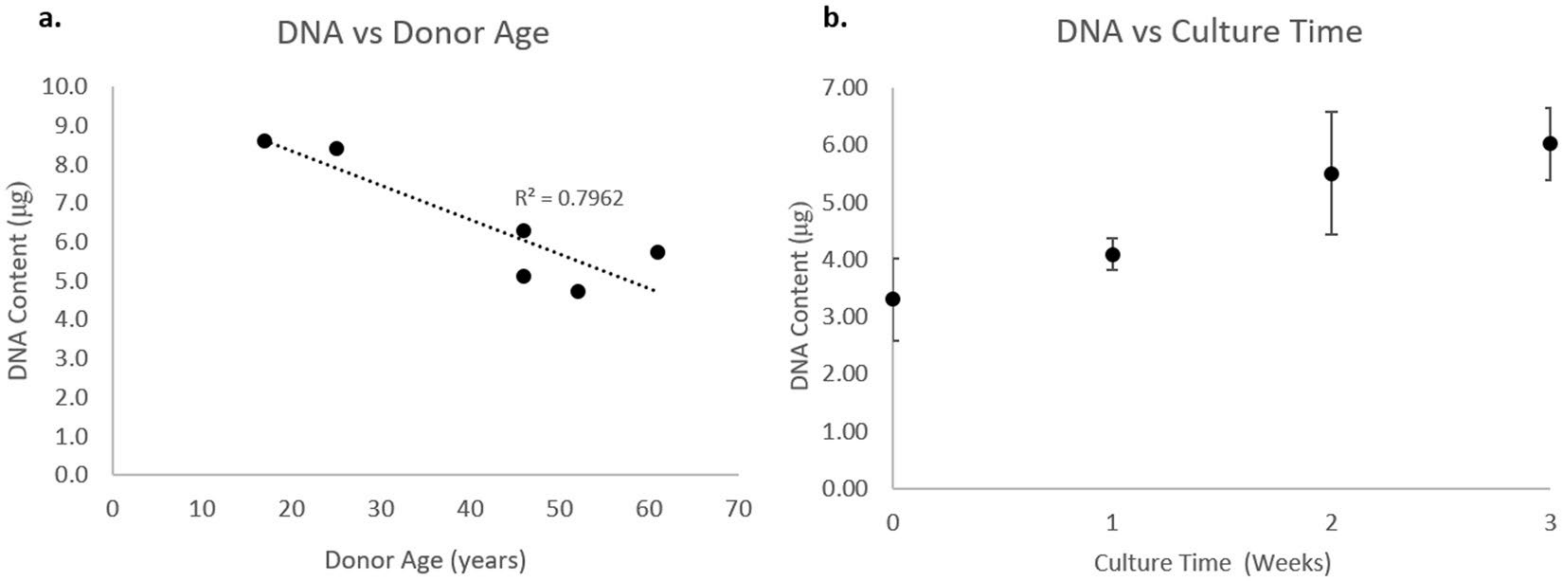
Left: Plot of constructs DNA content versus donor age. Density significantly decreased with increasing age p < 0.05. Right: Plot illustrating the increase in DNA over time in culture on Chondro-Gide scaffolds.

After cells were isolated from 6 donors, the cells were expanded *in vitro* to passage 2 (P2). The cells went through an average of 4.0 ± 0.3 doublings prior to cell seeding on Chondro-Gide scaffolds. After 3 weeks of normoxic culture in 3D spinner flasks, the total GAG content per construct was measured using the dimethyl-methylene blue (DMMB) assay. GAG content varied substantially between donors with an average GAG content of 154 ± 67 µg per construct (range 50–260 µg). When GAG content was normalized to DNA content (GAG/DNA) in each scaffold, the average GAG/DNA also content varied considerably with an average of 23.3 ± 7.7 µg/µg (range 10.5–31.1). Neither total GAG nor the GAG/DNA was significantly correlated with donor age. Total DNA content in the constructs was significantly and negatively correlated with age, with decreasing total DNA for increasing age, R^2^ = 0.80, p < 0.05 (Fig. 2). One independent replicate was excluded from the analysis as an outlier due to very low DNA content. A pilot study was conducted on three donors (all male, ages: 24, 25, 31) where DNA content was determined on the day of initial seeding (day 0 controls) and each week for 3 weeks using biological replicates. Cells underwent an average of 0.9 doublings after they were seeded on the scaffolds in chondrogenic media (DNA content increased from 3.3 ± 0.7 µg to 6.0 ± 0.6 µg) (Fig. 2).

**Figure 3:**
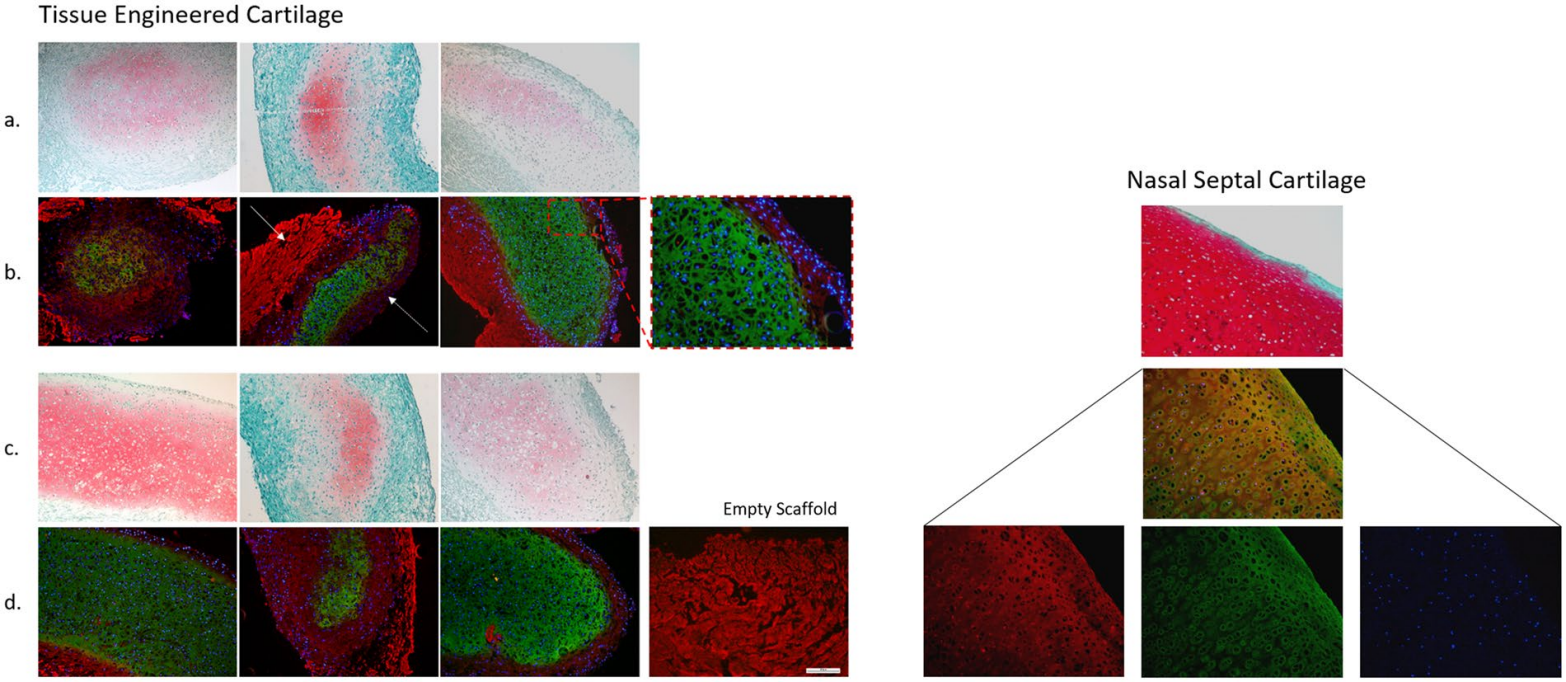
Left: Panel image showing all six donors’ histology (**a**,**c** Fast green, Safranin O and haematoxylin staining showing the distribution of the collagen, GAG and cells respectively. Immunofluorescence (**b**,**d** corresponding to the histology image immediately above) for collagen type I and type II with DAPI for cell nuclei. Right, native septal cartilage histology and immunofluorescence for comparison. Scale bar on bottom right image is 200 μm. The solid white arrow in B2 indicates the Chondro-Gide scaffold fluorescence for collagen I while the dashed arrow indicates newly formed collagen I produced by the cells and are clearly distinguishable from each other. Breakout image is a 2x zoom if the scaffold surface demonstrating the discrete change from collagen I to collagen II fluorescence.

Safranin O staining for the detection of glycosaminoglycan (GAG) qualitatively corresponded with DMMB measurement. Construct histology was evaluated using the Bern Score for *in vitro* TE cartilage^16^: scores can range from zero (poor quality) to nine (excellent quality) with 3 sub-scores for Safranin O staining, cell density, and cell morphology. In our samples, scores ranged from 5–9, with a mean of 6.6 ± 1.4. Bern scores correlated positively with total GAG content in the scaffolds, R^2^ = 0.80, p < 0.05. Safranin O was not seen at the surfaces of the tissues but was diffuse throughout five of the six constructs (Fig. 3). Native nasal cartilage stained very intensely for Safranin O, with the exception of the surface layer where there was no staining. Immunofluorescence imaging also corresponded well with the histological staining. Collagen II was seen to correlate with the presence of Safranin O staining. Collagen I was observed in regions that were negative for Safranin O staining. Negative controls (secondary antibody only) showed no non-specific binding of the secondary antibodies. However, the empty scaffold control showed strong binding with our primary antibody to collagen I. Empty scaffolds demonstrated diffuse collagen I immunofluorescence in a distinctive pattern of large bundles (Fig. 3). Collagen I immunofluorescence in the newly formed cartilage on the scaffolds was less intense and did not appear to form bundles. The neo-tissue and scaffold immunofluorescence patterns are distinctly different, and thus could be easily distinguished. Immunofluorescence in the native tissue showed co-localization of collagen I and II. Strong collagen II immunofluorescence was observed in the extracellular space that surrounds the peri-cellular matrix. Fluorescence imaging also demonstrated columnar patterns in the cells that were perpendicular to the septal surfaces. Comparison of the native tissue to engineered cartilage also illustrated higher cell density and less distinct lacunae structures in the engineered cartilage compared to native tissue.

**Figure 4:**
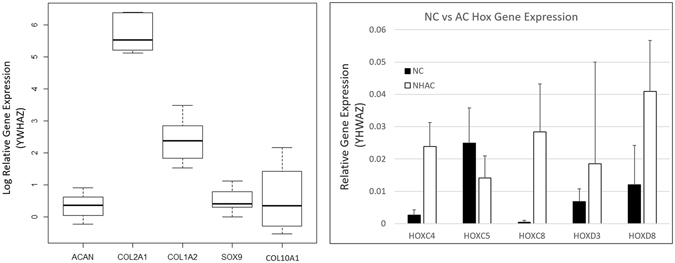
Left: Box plot of the natural log of the gene expression for *ACAN*, *COL2A1*, *COL1A2*, *SOX9* and *COL10A1* from objective 1. Gene expression showed a lognormal distribution with Shapiro Wilk p > 0.05 and non-skew boxplots. Relative gene expression of the Hox genes *HOXC4*, *HOXC5*, *HOXC8*, *HOXD3* and *HOXD8* against the reference gene *YHWAZ* for nasal chondrocytes (NC) vs normal human articular cartilage (NHAC). Error bars ± SD.

Gene expression via reverse transcription quantitative polymerase chain reaction (RT-qPCR) demonstrated variability between donors (Fig. 4). Collagen II was most upregulated in the TE engineered constructs which corresponds with immunofluorescence findings. We evaluated the family of homeobox (Hox) genes between NC and articular chondrocytes (AC) as they have been shown to display different Hox gene expression due to their different developmental lineage. This gene expression has been demonstrated to be associated with the plasticity of NC^17^. Expression of the Hox genes was not statistically different between NC and AC due to small sample size of AC (n = 3), however there was a trend for *HOXC4*, *HOXC8* and *HOXD8* to be expressed at higher levels in AC than NC cells (Fig. 4). Further, *HOXC5* and *HOXC8* were significantly and negatively correlated in NC (R^2^ = 0.92, p = 0.0027, adjusted significance level p < 0.005 for multiple comparisons).

### Objective 2: Strategies to Mitigate Variability

**Figure 5:**
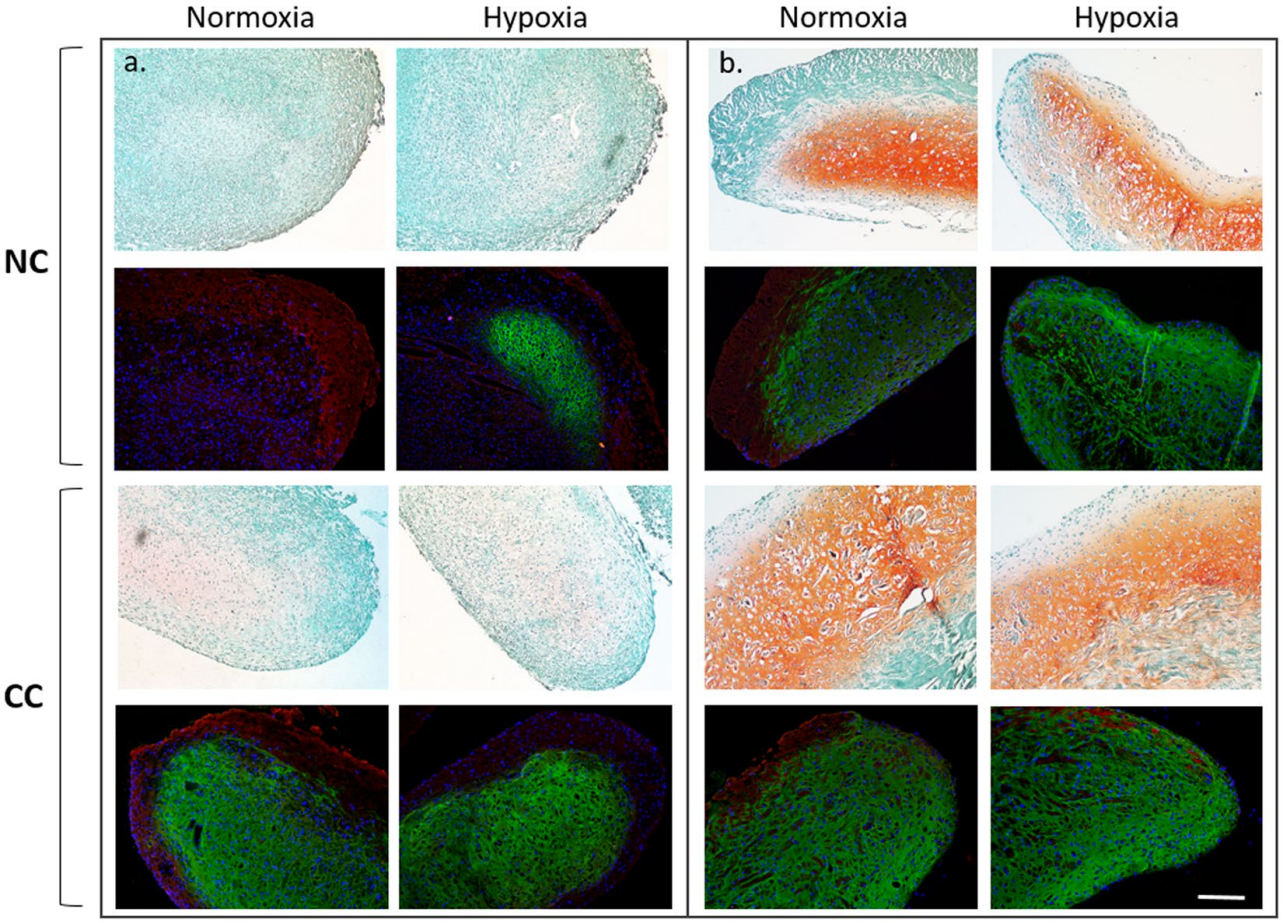
Effect of hypoxia and co-culture on tissue quality. (**a**). Top: Histological and immunofluorescence images for the worst donor (left, Bern Score = 1) in both normoxia and hypoxia. Bottom: Tissue generated with chondrocytes from the same donor co-cultured with BM-MSC in normoxia and hypoxia (**b**). Top: Histological and immunofluorescence images for the best donor (left, Bern Score = 8) Bottom: Tissue generated with chondrocytes from the same donor co-cultured with BM-MSCs. Fast green, Safranin O and haematoxylin staining showing the distribution of the collagen, GAG and cells respectively. Immunofluorescence images, red fluorescence is collagen I, green is collagen II and blue is DAPI for cell nuclei. Scale bar is 200 µm.

**Figure 6:**
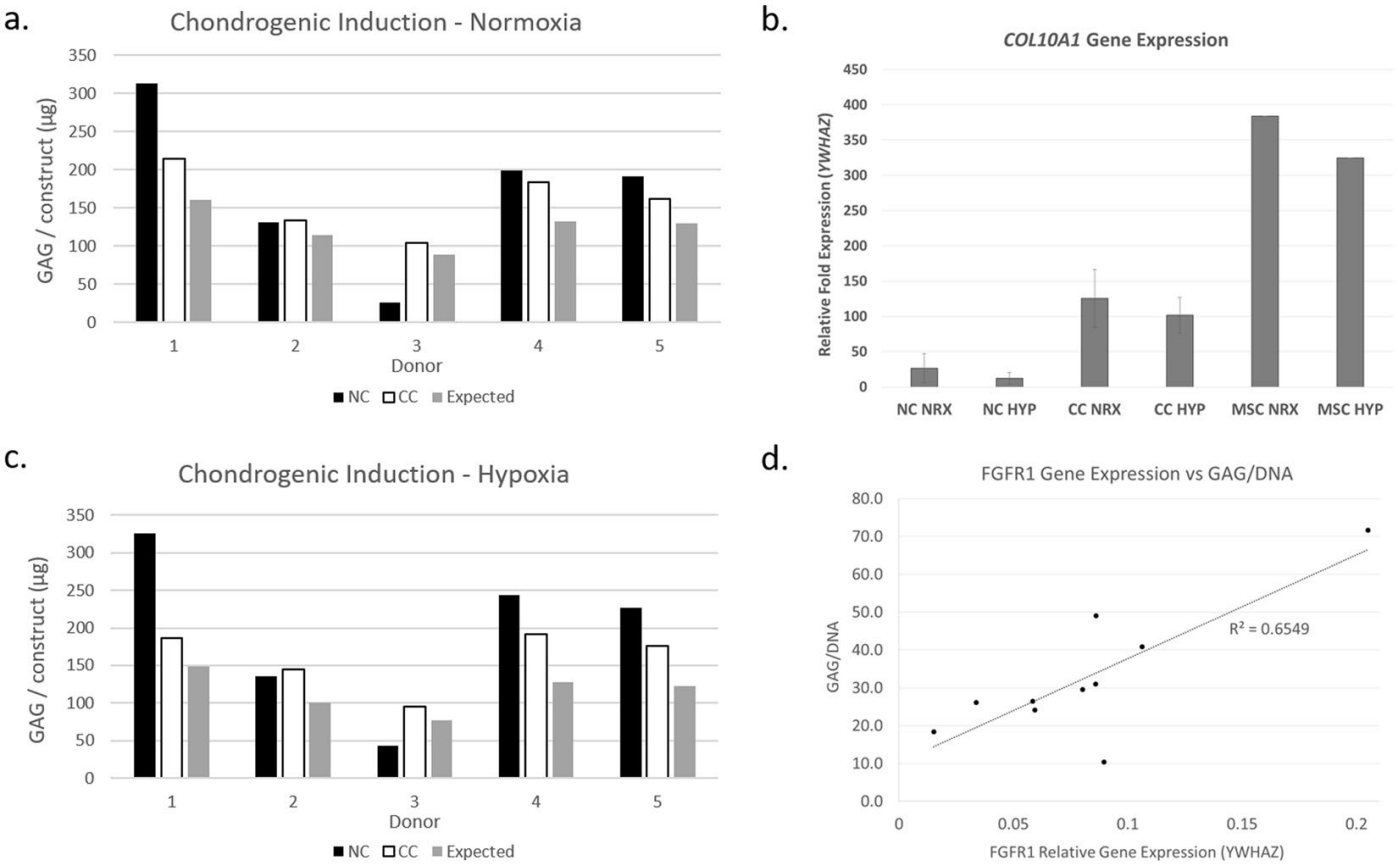
(**a**,**c**) Interaction index identifying chondro-induction in co-culture for both normoxia (top) and hypoxia (bottom) for all 5 donors. Black bars are nasal chondrocytes, white bars are co-culture and grey is the expected value from the NC and BM-MSC without any interaction. CC values higher than expected values indicated a chondro inductive effect. (**b**) Relative gene expression for *COL10A1* for the groups NC, CC and MSC in normoxia (NRX) and hypoxia (HYP) normalized against *YHWAZ*. (**d**) Regression of GAG/DNA vs gene expression of *FGFR1*, showing a positive and significant correlation, R^2^ = 0.66, adjusted p < 0.05.

The tissue received was of inconsistent quality with three donors exhibiting normal gross appearance and two which had a more fibrous tissue quality prior to digestion. The variability in initial tissue quality appeared to influence the quality of tissue generated in our tissue engineered constructs. The two donors with the initial fibrous appearance benefited from co-culture (CC) with BM-MSCs, with more cartilage-like histological staining and increased collagen II fluorescence (Fig. 5). The remaining 3 donors resulted in similar or decreased Safranin O intensity and total GAG for co-culture compared to nasal chondrocytes alone. Bern Scores for NC ranged from 1–8 in both normoxia (NRX) and hypoxia (HYP); mean 5.5 ± 2.7, 5.9 ± 2.8 respectively, while CC ranged from 4.5–6 in NRX and 4–6 in HYP (mean 5.1 ± 0.65, 4.7 ± 0.84 respectively). Bern Scores were significantly positively correlated with total GAG in TE cartilage (R^2^ = 0.81, p < 0.001). Total GAG amounts varied widely in NC TE cartilage; NRX range 26–312 μg (mean 172 ± 105 μg) HYP range 43–326 μg (mean 195 ± 109 μg). In both NRX and HYP conditions there was a statistically significant chondro-inductive effect from co-culture with BM-MSC (Interaction Index NRX: 95% confidence interval (CI) [1.18, 1.35] and HYP: 95% CI [1.27–1.48], where >1 defines a chondro-inductive effect, Fig. 6). The Interaction Index was not statistically different between normoxia and hypoxia conditions (p > 0.05). There were no significant differences in DNA content in either NRX vs HYP or in NC vs CC.

For objective 2, gene expression was completed on four of the five donors due to limited RNA extraction from one donor. Expression of collagen X (*COL10A1*) was greater in CC than in NC (p < 0.05, NRX grouped with HYP n = 8, Fig. 6). However, *COL10A*1 was downregulated in co-culture compared to BM-MSC values for all donors measured in both oxygen conditions.

To evaluate the gene expression of the fibroblast growth factor receptors (*FGFR1-4*), extracellular matrix (ECM) proteins: collagen II (*COL2A1*), aggrecan (*ACAN*), collagen X (*COL10A1*), collagen I (*COL1A2*) and transcription factor *SOX9*; cartilage constructs from the NRX condition were pooled from objective 1 and objective 2 to increase the sample size (n = 10, the worst donor from objective 2, that showed little chondrogenic capacity, Bern Score = 1 was not included due to limited RNA extraction). If gene expression exhibited a lognormal distribution, the data were log transformed. The resulting transformed data followed a normal distribution for *COL2A1*, *COL1A2*, *COL10A1*, *ACAN*, *SOX9* and *FGFR2*, Shapiro-Wilk test (p > 0.05). *FGFR1*, *FGFR3*, *FGFR4* were normally distributed without transformation. Pearson’s correlations were determined for the FGFR family against GAG and GAG/DNA to determine their influence on tissue quality. ECM molecules were regressed against each other in the cartilage constructs. Collagen II was positively and significantly correlated with aggrecan and collagen X expression (R^2^ = 0.74, R^2^ = 0.74 respectively, adjusted p < 0.05). There was also a significant positive correlation between *FGFR1* and GAG/DNA (R^2^ = 0.66, adjusted p < 0.05, Fig. 6). No other statistically significant correlations were observed.

### Objective 3: Phenotypic Stability

**Figure 7:**
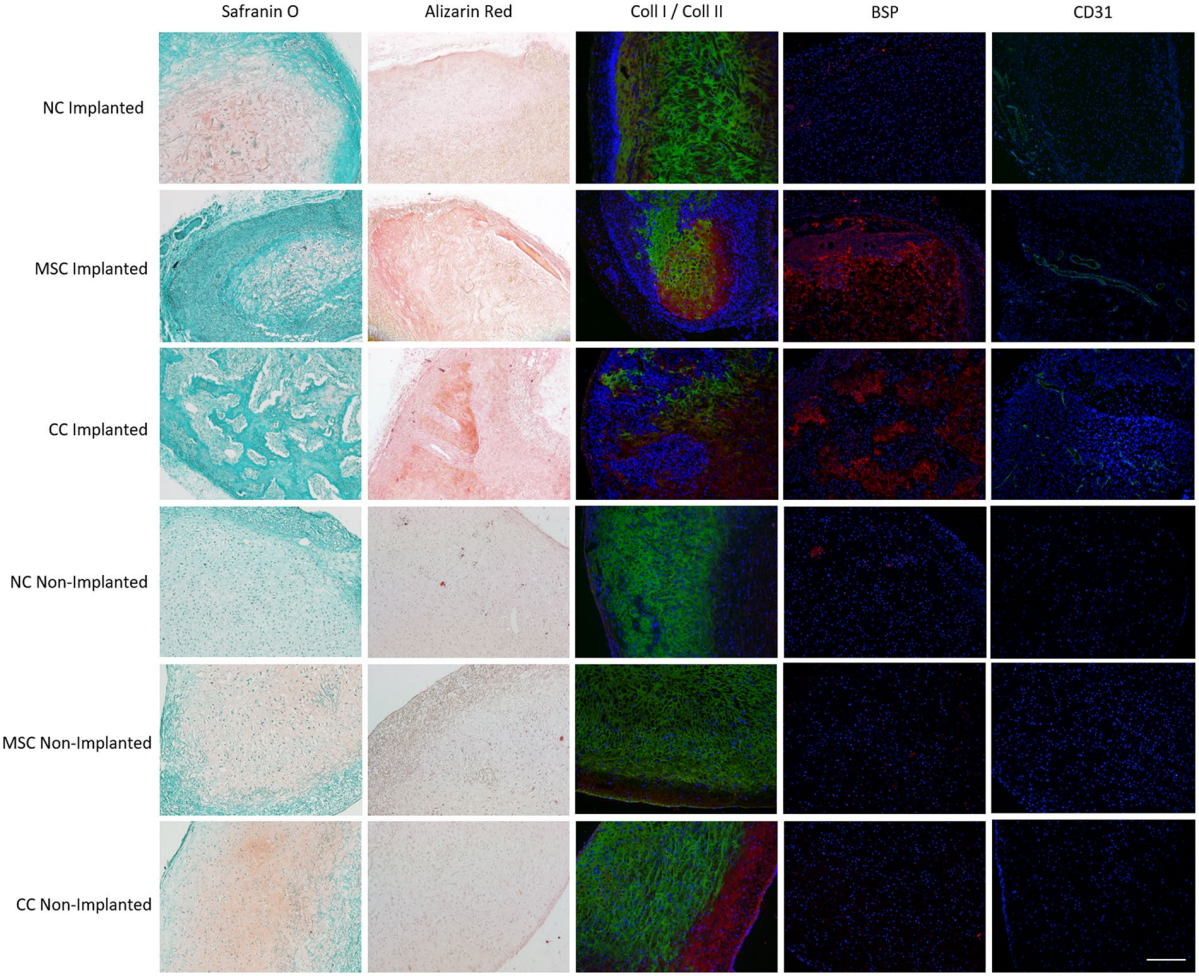
Panel image illustrating the phenotypic stability characteristics of the tissue culture strategies for one donor in normoxic conditions (similar patterns were seen in hypoxia and in Donor 2). From left to right, Fast Green/Safranin O (GAG distribution), Alizarin Red (calcification), immunofluorescence for collagen I and collagen II, bone sialoprotein (BSP), and CD31 - in all fluorescence images DAPI for cell nuclei. The NC condition resulted in no calcification, normal collagen fluorescence and minimal, punctate BSP fluorescence similar to non-implanted controls. There was some vascular invasion as seen by positive CD31 fluorescence but only on the periphery of the construct and not in the cartilaginous region. Both MSC and CC groups demonstrated, very little GAG staining, positive Alizarin staining for calcification, diffuse BSP fluorescence and pervasive CD31 fluorescence throughout the constructs. Scale bar 200 μm.

To determine the phenotypic stability of the constructs generated with the strategies used above; cartilage constructs were cultured for 3 weeks *in vitro*, then implanted subcutaneously in nude mice for 5 weeks. Cartilaginous TE scaffolds integrated into the nude mice with no signs of infection/rejection. The scaffolds incorporated well and contained very high cell density compared to non-implanted controls, indicating invasion of mouse cells. Implanted scaffolds had significantly higher DNA content than non-implanted controls (DNA 11.1 ± 4.7 μg vs. 2.5 ± 0.8 μg respectively, p < 0.05) and the 3 week cultured constructs from objective 2 (11.1 ± 4.7 μg vs. 4.7 ± 1.0 μg respectively p < 0.05). The 3-week cultured constructs also contained significantly higher DNA than non-implanted controls (p < 0.05), indicating that between 3 weeks and 8 weeks the cells of the TE cartilage begin to die in static culture. GAG content was significantly greater in non-implanted versus implanted constructs (94.0 ± 30.7 μg vs 58.8 ± 21.5 μg, p < 0.05). Upon digestion of the constructs in proteinase K, it was observed that all implanted constructs cultured using BM-MSC (both BM-MSC and CC groups) contained undigested tissue. Through histological and immunofluorescence imaging it was observed that bony tissue had formed in the constructs that contained BM-MSC. Alizarin Red staining indicated calcified tissue formation in both CC and BM-MSC groups but not in the NC group. Bone sialoprotein (BSP) was also present throughout the BM-MSC containing scaffolds but only demonstrated punctate pericellular fluorescence in the NC group; similar to the non-implanted controls (Fig. 7). Invasion of blood vessel was also observed using CD31 immunofluorescence. All implanted constructs showed some degree of vascularization, however in the NC group, the vessels were restricted to non-cartilaginous regions on the periphery of the scaffold (Fig. 7); most likely in the non-porous side of the Chondro-Gide scaffold or the murine connective tissue surrounding the implant. Implanted NC constructs stained positively from Safranin O and collagen II while co-culture and BM-MSC groups had qualitatively less collagen II and no observable Safranin O. In contrast, the non-implanted controls were positive for both Safranin O and collagen II; indicating a phenotypic shift after implantation.

## Discussion

We have demonstrated an ability to develop nasal tissue engineered constructs using expanded nasal chondrocytes on a commercially available, clinically approved scaffold (Chondro-Gide). Further, we have compared the Safranin O staining and distribution of collagen I and collagen II of our constructs to native nasal septal cartilage. We have also evaluated the gene expression levels of several molecules of interest in cartilage tissue engineering (the majority of which are characteristic extracellular matrix molecules) including: aggrecan, collagen I, collagen II, collagen X and the transcription factor, *SOX9* in order to better understand normal donor dependent variability in TE nasal cartilage. While there were no apparent effects of donor age on the quality of tissue produced, there was a statistical decrease in the amount of DNA in the constructs with increasing donor age. This may indicate that the cells from younger donors have higher proliferative capacity in our 3D scaffolds, than cells from older donors. Our pilot data demonstrated that DNA content increased almost twofold over three-week culture period. Histological and immunohistochemical analyses demonstrated high cell density in our constructs after 3 weeks in culture. Nasal chondrocytes exhibit increased capacity for cell proliferation and chondrogenic capacity when expanded *in vitro* compared to articular chondrocytes^17^. The differential expression of Hox genes which we have also demonstrated here is hypothesised to play a role in this behaviour^17, 18^.

Gene expression analysis suggests a nasal cartilage phenotype after expansion to passage 2 (average 4.0 ± 0.3 doublings). Aggrecan and collagen II were both upregulated compared to the housekeeping gene *YWHAZ*. Moreover, the minimal expression of *HOXC4* and *HOXD8* correspond with previous findings that demonstrated that these genes are not expressed at detectable levels in nasal chondrocytes when compared to articular cartilage^17^. That study demonstrated non-detectable levels of both *HOXC4* and *HOXD8* against the reference gene *GAPDH*. We have seen detectable, but down-regulated expression of these two genes against our reference gene *YWHAZ*. As *GAPDH* is associated with glycolysis, which is the dominant metabolic pathway in cartilage^19^, it may be upregulated compared to *YWHAZ* in chondrocytes, thus resulting in a lower relative expression in the previous study than we have observed. Overall, we have observed a nasal cartilage-like gene expression profile which is also supported by the histological and biochemical analyses. Surprisingly, expression of *FGFR1* was positively correlated with the cartilage phenotype in nasal chondrocytes. In articular cartilage, *FGFR3* has been shown to correlate with chondrogenic capacity^20^. Further, fibroblast growth factor 18 (FGF-18) is a known anabolic factor that acts through *FGFR3* ^21^ in articular cartilage. *FGFR1* has been shown to be associated with the progression of cartilage degradation in mice^22^. However, previous work comparing nasal cartilage to articular cartilage has demonstrated that the craniofacial lineage of nasal cartilage results in differential gene expression when compared to articular cartilage^17^. These lineage differences may play a role in our findings regarding FGF-1. In Kallmann Syndrome, which results in cleft palate, *FGFR1* loss of function mutations have been identified in human populations and the FGFR-1c isoform is required for palate development^23^. Nasal septum deformity is often concomitant with cleft palate, which may be related to their common craniofacial lineage, but could also be a consequence of altered, asymmetrical mechanical and developmental cues. Further investigations are required to confirm the role of *FGFR1* in nasal cartilage development and its association with matrix quality.

We used two main strategies to attempt to increase the quality of tissue engineered cartilage; hypoxic culture and co-culture with bone marrow derived mesenchymal stromal cells. There were no significant differences in GAG or DNA content, nor in gene expression between normoxia and hypoxia. Malda *et al*. demonstrated a positive effect of oxygen tension on the redifferentiation and chondrogenic capacity of nasal chondrocytes^15^. However, it has also been demonstrated that normoxia conditions result in superior cell expansion and GAG production^24^. Whereas, Scotti *et al*. showed no significant effect of varied oxygen tension in expanded nasal chondrocytes^9^. Our results support the latter study with no substantial effect on cartilage tissue quality. These mixed results of oxygen tension on nasal cartilage may be the result of varying conditions during cell expansion and requires further examination. Co-culture did prove to be capable to improving poor quality donor tissue to a more cartilage like appearance. The biggest effect was observed brining a tissue with a Bern Score of 1, to 4.5 which is a dramatic increase tissue quality. All donors experienced a chondro-inductive effect, where more GAG was measured than would be expected based on the individual cell ratios alone. This paracrine effect is evident in expanded nasal chondrocytes, which had been only previously identified in non-expanded primary nasal chondrocyte co-culture^12^. This synergistic effects of co-culture of BM-MSCs with NCs was observed here even after approximately 5 population doublings of NCs. Lopa *et al*. previously demonstrated no positive effect of co-culture with adipose derived MSCs^25^. That study used cryogenically preserved cells and had expanded the cells with the use of transforming growth factor- β1 (TGF-β1), which may have resulted in their negative finding. In this study, co-culture was also able to supress type X collagen gene expression relative to BM-MSC. Despite reducing the hypertrophic chondrocyte phenotype, co-culture still resulted in calcified tissue and blood vessel invasion after implantation in the nude mouse model. BM-MSCs constructs also showed induction of hypertrophy and subsequent calcification on Chondro-Gide scaffolds; a similar results was previously obtained with implantation of BM-MSCs in a pellet model^14^. A significant limitation of this work is that in objective 1, variability was assessed in only six donors. However, combining this with the five donors from objective 2 yields 11 donors that demonstrated a wide range in tissue quality (Bern score range: 1–9); which included very poor and excellent donors. The work of Fulco *et al*. showed large variability in only 5 donors within a relatively small age range (76–88 years)^11^. Another limitation of this study is that in both objectives 2 and 3, a single MSCs donor was used in combination with the various nasal chondrocyte donors. Therefore, despite the positive effects of co-culture on poor quality donor tissue, more work is required to determine whether cell hypertrophy and subsequent calcification can be supressed with the use of BM-MSCs. While the goal of this experimental design was to limit the variability in the system to only the variability in the NC, it may limit the generalizability of the results. There is significant heterogeneity in the chondrogenic and osteogenic capacity of cells generated from plastic adherence of bone marrow aspirates between patients, and also importantly, within a single patient’s aspirate^26^. The use of cell sorting strategies based on clonal screening of cell surface markers or gene expression may allow for the selection of a non-osteogenic subpopulations that has similar positive effects on tissue quality. Moreover, the use of alternate stem cells populations with lower osteogenic capacity such as synovial fluid derived stem cells^27^ may be another alternative strategy to reduce ectopic calcification *in vivo*.

In conclusion, we have demonstrated that *in vitro* culture expanded nasal chondrocytes are a viable source for the development of tissue engineered nasal cartilage. Further, culture of TE nasal cartilage can be conducted in either normoxic or hypoxic conditions with similar results. Finally, the inherent donor-to-donor variability in septal chondrocyte quality can be mitigated through the use of co-culture with BM-MSCs. However, further work is required to select an optimal stem cell source to generate phenotypically stable constructs to be considered as a viable option for nasal reconstruction.

## Methods

### Objective 1: Examination of Inter-subject Variability

#### Cell Isolation and Expansion

Nasal cartilage was received from surgically discarded material from 6 donors (5 male, age range (17–61), 1 female age 25) undergoing septoplasty and rhinoseptoplasty procedures. Experimental methods and tissue collection were with the approval of and in accordance the University of Alberta’s Health Research Ethics Board- Biomedical Panel (Study ID: Pro00018778). Cartilage was digested in 0.15% (w/v) type II collagenase (300 units/mg Worthington, Lakewood, NJ, USA) in standard media; Dulbecco’s Modified Eagle Medium (DMEM, Sigma) supplemented with heat inactivated FBS (5% v/v; Gibco), 100 U/ml penicillin and streptomycin with L-glutamine (2 mM), HEPES (0.1 M) (All from Life Technologies, Mississauga, ON, Canada) in a shaker at 37 °C for 22 hours. The tissue digests containing matrix released chondrocytes was then passed through a 70 µm nylon strainer. Cells were washed three times in sterile phosphate buffered saline (PBS; Sigma), then resuspended in standard media and plated in T150 tissue culture flasks in a normoxic humidified incubator (21% O_2_, 5% CO_2_). After 24 hours the cells were trypsinized and counted using a standard counting protocol with trypan blue and a hemocytometer^28^. Cells were then plated at a density of 10^4 ^cells/cm^2^ in our standard medium (DMEM Complete) supplemented with TGF-β1 (1 ng/ml), Prospec, Ness Ziona, Israel) and fibroblast growth factor 2 (FGF-2; (5 ng/ml), Prospec), which has been demonstrated to increase the proliferation and post-expansion redifferentiation capacity of chondrocytes^13^. The media was changed twice weekly and cells were passaged (1:2) after they reached confluency until passage 2 (P2). At the end of P2, the cells were counted again and the population doublings were calculated; log_2_ (population _final_/population _initial_). Two passages were used as it results in expansion of the cell population to produce a tissue construct of sufficient size from a small donor biopsy^11^.

#### Cell Seeding and Tissue Culture

After counting, the cells were resuspended in serum free chondrogenic culture media (DMEM, 100 U/ml penicillin and streptomycin with 2 mM L-glutamine (Life Technologies, all), 100 mM HEPES, insulin-transferrin-selenium (ITS) +1, 0.1 µM dexamethasone, 0.1 mM ascorbic acid 2-phosphate and 0.1 mM L-proline. Cells were seeded on the porous side of Chondro-Gide scaffolds (Geistlich Pharma, Wolhusen, Switzerland). Scaffolds (6 mm diameter × 2 mm) were seeded with 0.5 × 10^6^ cells suspended in 20 µl of chondrogenic media (density 8.8 × 10^6^ cells/cm^3^), in a 24-well plate. The scaffolds were incubated for 15 minutes, then an additional 100 µl of media was added to the well and incubated for 45 minutes, to promote cell adhesion. Finally, for each donor all replicate scaffolds were placed in a spinner flask (50 or 100 mL) (Bellco Glass, Inc. Vineland NJ, USA) and 40 mL of chondrogenic media added. The spinner flasks were placed on a multipoint magnetic stirrer (Thermo Scientific), inside the incubator and rotated at 80 RPM. The impeller was positioned such that only the bottom half was immersed in the media to reduce the amount of rotation induced in the media. The spinner flask was used to increase nutrient transport in the scaffolds by inducing a fluid shear on the surface. The media was changed weekly for 3 weeks of tissue culture. After the culture period, the constructs were prepared for analysis including histology, immunofluorescence imaging and RT-qPCR.

#### Histology/Immunofluorescence

Tissue engineered constructs were fixed in 10% wt/vol neutral buffered formalin for 48 hours at 4 °C. The samples were then processed into paraffin wax and embedded. Sections were cut at 7 µm thickness. Nasal septal cartilage from 2 donors was harvested and prepared similarly. For histological analysis, sections were stained using a 0.1% wt/vol Safranin O and counter stained with 1% wt/vol fast green. Sections were scored using the Bern Score for *in vitro* tissue engineered cartilage^16^. The distribution of types I and II collagen were evaluated using immunofluorescence imaging. Sections were deparaffinized and rehydrated. Antigen retrieval was conducted with Protease XXV (Thermo Scientific) for 30 minutes at room temperature and hyaluronidase (H6254, Sigma) for 30 minutes at 37 °C. Sections were then blocked with 5% wt/vol bovine serum albumin in PBS for 30 minutes at room temperature. Labeling of collagen I and collagen II was completed using rabbit anti-collagen I, 1:200 dilution (CL50111AP-1, Cedarlane) and mouse anti-collagen II, 1:200 dilution (II-II6B3, Developmental Studies Hybridoma Bank, University of Iowa, USA) respectively and incubated overnight at 4 °C. Secondary labelling using goat anti-mouse IgG, Alexa Fluor 488, 1:200 (ab150117, Abcam) and goat anti-rabbit IgG, Alexa Fluor 594, 1:200 (ab150080, Abcam) incubated at room temperature for 1 hour. Slides were mounted using Everbrite Hardset Mounting Medium with DAPI (Biotium) and imaged using a Nikon Ti-S Microscope with DS-U3/Fi2 Color CCD camera using FITC and Texas Red filters.

#### Glycosaminoglycan (GAG) and DNA Quantification

Cultured nasal cartilage scaffold constructs were rinsed with PBS and frozen at −80°C. Scaffolds were then thawed and digested in Proteinase K (1 mg/ml, Sigma) for 16 hours at 56 °C. A 1,9, dimethylmethylene blue (DMMB) binding assay was used to quantify GAG content; using chondroitin sulfate (Sigma-Aldrich) as the standard. The CyQUANT Cell Proliferation Assay Kit (Life Technologies) was used to quantify the DNA content in the scaffolds. The supplied bacteriophage λ DNA was used as the standard for that assay.

#### Reverse transcription quantitative real-time polymerase chain reaction (RT-qPCR)

RT-qPCR was conducted as described previously^29^. Briefly, scaffolds were placed in TRIzol Reagent (Life Technologies) and frozen at −80 °C. Samples were later thawed at room temperature. Total RNA was extracted from the nasal cartilage constructs using TRIzol after grinding them with a pestle. Total RNA (100 ng) was reverse-transcribed to cDNA using GoScript Reverse Transcription System (Promega Corp.) primed in the presence of oligo(dT) primers (1 µg). Quantitative PCR was conducted on a DNA Engine Opticon I Continuous Fluorescence Detection System (Bio-Rad Laboratories, Hercules, CA, USA) using HotGoldStar Taq polymerase and SYBR Green detection (Eurogentec North America Inc., San Diego, CA, USA). The primer sequences were developed using the National Center for Biotechnology Information (NCBI) database and designed using Primer Express software (Applied Biosystems, Foster City, CA, USA). Primer sequences were virtually tested using Basic Local Alignment Search Tool (BLAST, NCBI) prior to ordering for oligo synthesis. Designed primer sequences are shown in Table 1.

**Table 1:** RT-qPCR Primer Sequences.

Gene	Accession #	Forward	Reverse
*ACAN*	M_55172	AGGGCGAGTGGAATGATGTT	GGTGGCTGTGCCCTTTTTAC
*COL1A2*	NM_000089	TTG CCC AAA GTT GTC CTC TTC T	AGC TTC TGT GGA ACC ATG GAA
*COL2A1*	NM_033150	CTG CAA AAT AAA ATC TCG GTG TTC T	GGG CAT TTG ACT CAC ACC AGT
*COL10A1*	X60382	GAAGTTATAATTTACACTGAGGGTTTCAAA	GAGGCACAGCTTAAAAGTTTTAAACA
*SOX9*	Z46629	CTTTGGTTTGTGTTCGTGTTTTG	AGAGAAAGAAAAAGGGAAAGGTAAGTTT
*YWHAZ*	NM_003406	TCTGTCTTGTCACCAACCATTCTT	TCATGCGGCCTTTTTCCA
*HOXC4*	NM_014620.5	ACCGTCGCATGAAATGGAA	TGCTGACCTGACTTTGGTGTTG
*HOXC5*	NM_018953.3	GGTGCAGGCATCCAGGTACT	CGGTGGGAAAGTGATGCTTAA
*HOXC*8	NM_022658.3	AACCCGTGCTCGCTTAGCT	GCCTCGTAGCCATAGAATTTGG
*HOXD3*	NM_006898.4	GGAGCTTCCTGAGTGCACAAT	CCTCCAAACAGTCCTGGGTTT
*HOXD8*	NM_019558.3	GGAATTTCTTTTTAACCCCTATCTGA	GCTAGGGCGTGGGAAACC
*FGFR1*	NM_023110.2	AACCTGACCACAGAATTGGAGGCT	ATGCTGCCGTACTCATTCTCCACA
*FGFR2*	NM_000141.4	TGATGGACTTCCTTATGTCCGCGT	AGCGTCCTCTTCTGTGACATTGGT
*FGFR3*	NM_000142.4	ACCAATGTGTCTTTCGAGGATGCG	AGAGCACGCAGCTTGTCACATAGA
*FGFR4*	NM_002011.3	ATGGAACTGGTGTGCTCAAGAAGC	TTCACATGTCCTCCGACCAACACA

Gene expression levels were normalized to the housekeeping gene *YWHAZ* using to 2^−ΔΔC(T)^ method^30, 31^. A family of Hox genes including *HOXC4*, *HOXC5*, *HOXC8*, *HOXD3* and *HOXD8* were evaluated as they have been shown to be expressed differentially in craniofacial derived cartilages relative to joint articular cartilage (especially of the knee joint) and are associated with plasticity in the cell phenotype^17^. Hox gene expression in TE nasal cartilage, was compared against normal human articular chondrocytes obtained from cadaveric donation. The cells were primary, monolayer controls, isolated from normal articular cartilage as described above, from 3 male donors (ages 47, 61, 62). The family of fibroblast growth factor receptors (FGFRs) were also assessed to explore their correlation with cartilage quality; as *FGFR3* has been linked with MSCs differentiation and mature articular chondrocyte cartilage matrix production through activation with fibroblast growth factor 18 (FGF-18)^21, 32^.

### Objective 2: Strategies to Mitigate Variability

Nasal chondrocytes were isolated and expanded as above (n = 5, 3 male age 22–32, 2 female age 32 and 42). BM-MSCs were isolated from the iliac crest of 1 donor (female, age 58) during an orthopaedic procedure obtained as surgical discard material. Cells were then expanded in the presence of 5 ng/ml FGF-2 for a total of two passages as previously described^33^. Cells were expanded under normoxic conditions (21% O_2_) to passage 2 and the population doublings calculated. A single bone marrow donor was used for both objectives 2 and 3 to limit the donor-to-donor variability for these studies to only the nasal chondrocytes.

Cells were then seeded on Chondro-Gide at a density 0.300 × 10^6^ cells per scaffold (6 mm diameter × 2 mm thickness, 5.3 × 10^6^ cells/cm^3^). The lower cell density was used in this objective do to reduce the number of BM-MSC required, however was still within the optimal seeding density of 5–10 × 10^6^ cells/cm^3^ previously demonstrated in our lab^34^. The cells were seeded in one of 3 conditions, nasal chondrocytes only (NC), BM-MSCs only (MSC) or in a co-culture of 3:1 BM-MSC to nasal chondrocytes (CC). These seeded scaffolds were cultured in either normoxia (NRX, 21% O_2_) or in hypoxia (HYP, 3% O_2_) incubators in 24 well plates for 3 weeks, with media changed twice a week.

Immunofluorescence, histology and GAG and DNA quantification were completed as in objective 1. For co-culture scaffolds the interaction index was calculated to determine any chondro-induction effects^12^. Interaction index is defined by eq. 1:

1 $$\begin{array}{ccc}{\rm{I}}{\rm{n}}{\rm{t}}{\rm{e}}{\rm{r}}{\rm{a}}{\rm{c}}{\rm{t}}{\rm{i}}{\rm{o}}{\rm{n}}\,{\rm{I}}{\rm{n}}{\rm{d}}{\rm{e}}{\rm{x}} & = & \frac{GA{G}_{expected}}{GA{G}_{measured}}\\ {\rm{w}}{\rm{h}}{\rm{e}}{\rm{r}}{\rm{e}}:GA{G}_{expected} & = & 75{\rm{ \% }}\ast GA{G}_{MSC}+25{\rm{ \% }}\ast GA{G}_{NC}\end{array}$$

RT-qPCR was conducted as above, however the *HOX* family of genes were not evaluated.

### Objective 3: Phenotypic Stability

To evaluate the clinical feasibility of the strategies implemented in objective 2, cartilage constructs were implanted into athymic nude CD-1 mice (n = 9, Charles River, Wilmington, USA). Cells were isolated from 2 nasal cartilage donors (1 male, age 19; 1 female age 31) and 1 BM-MSC donor (male age 51) cells seeded on Chondro-Gide scaffolds as in Objective 2 (NC, MSC, CC in NRX and HYP). After 3 weeks of culture, half of the samples were implanted subcutaneously in nude mice. All procedures were approved and undertaken under the auspices of and in accordance with the University of Alberta Animal Care and Use Committee. Four constructs were implanted subcutaneously (2 cranial and 2 caudal) on the back of each mouse using a small incision that was closed by sutures and cyanoacrylate tissue adhesive. After 5 weeks of implantation, the constructs were recovered with the animals under anesthesia and the animals were subsequently euthanized. No adverse events were recorded with any of the mice and all constructs were recovered after the 5 week implantation period. After removal from the animal, any excess mouse connective tissue was carefully removed from the sample with a scalpel. The other half of the constructs were maintained in incubator culture in DMEM Complete without additional growth factors for 5 weeks (8 weeks total culture) to serve as a non-implanted control.

All samples were processed as described previously for GAG and DNA content. For histology and immunohistological analyses, to facilitate sectioning, the constructs were partially decalcified using 10% wt/vol EDTA and 0.1% wt/vol formalin (Sigma-Aldrich) for 1 weeks at 4 °C. Following decalcification, the samples were fixed in 10% wt/vol formalin for 24 hours and paraffin embedded as described above. To assess the phenotypic stability of these samples, bone formation was assessed. Alizarin Red staining was completed with deparaffinization as above, followed by Alizarin Red stain (2% wt/vol, pH 4.1–4.3, Sigma-Aldrich) for 2.5 minutes. Immunofluorescence imaging was completed to assess vascular invasion (CD31/PECAM Biotinylated Antibody 1:200, R&D Systems BAF3628 with Alexa Flour 488 Streptavidin secondary antibody, S32354, Life Technologies) and the bone associated protein, bone sialoprotein (BSP, Abcam, ab195426 1:200 with goat anti-rabbit IgG, Alexa Fluor 594, 1:500). Antigen retrieval was performed as in objective 1.

#### Statistics

Shapiro-Wilkes tests were conducted to determine the normality of the data. For non-parametric data, regression analysis was conducted using the Spearman’s Rank correlation test. For normally distributed data Pearson’s correlation was calculated. Between groups comparisons were made using t-tests or Wilcoxon sign-rank tests and corrected for multiple comparisons using the Holm-Bonferonni method^35^. Outliers were defined as values 1.5 interquartile ranges below the 1^st^ quartile or above the 3^rd^ quartile and reported when excluded from analyses. All statistics were calculated using Matlab Statistics Toolbox (The Mathworks Inc.).
