# Author Correction: Strategies to Mitigate Variability in Engineering Human Nasal Cartilage

**DOI:** 10.1038/s41598-019-53740-y

**Published:** 2019-11-14

**Authors:** Stephen H. J. Andrews, Melanie Kunze, Aillette Mulet-Sierra, Lynn Williams, Khalid Ansari, Martin Osswald, Adetola B. Adesida

**Affiliations:** 1 Laboratory of Stem Cell Biology and Orthopaedic Tissue Engineering, Divisions of Orthopaedic Surgery and Surgical Research, Department of Surgery, University of Alberta, Li Ka Shing Centre for Health Research Innovation, Edmonton, AB Canada; 2 Division of Otolaryngology-Head and Neck Surgery, Department of Surgery, University of Alberta Hospital, Edmonton, AB Canada; 3 Institute for Reconstructive Sciences in Medicine (iRSM), Misericordia Community Hospital, Edmonton, AB Canada

Correction to: *Scientific Reports* 10.1038/s41598-017-06666-2, published online 26 July 2017

This Article contains errors in Table 1 where the RT-qPCR Primer Sequences for the following genes,

**Table 1:** RT-qPCR Primer Sequences.

Gene	Accession #	Forward	Reverse
*ACAN*	M_55172	AGGGCGAGTGGAATGATGTT	GGTGGCTGTGCCCTTTTTAC
*COL1A2*	NM_000089	TTG CCC AAA GTT GTC CTC TTC T	AGC TTC TGT GGA ACC ATG GAA
*COL2A1*	NM_033150	CTG CAA AAT AAA ATC TCG GTG TTC T	GGG CAT TTG ACT CAC ACC AGT
*COL10A1*	X60382	GAAGTTATAATTTACACTGAGGGTTTCAAA	GAGGCACAGCTTAAAAGTTTTAAACA
*SOX9*	Z46629	CTTTGGTTTGTGTTCGTGTTTTG	AGAGAAAGAAAAAGGGAAAGGTAAGTTT
*YWHAZ*	NM_003406	TCTGTCTTGTCACCAACCATTCTT	TCATGCGGCCTTTTTCCA
*HOXC4*	NM_014620.5	ACCGTCGCATGAAATGGAA	TGCTGACCTGACTTTGGTGTTG
*HOXC5*	NM_018953.3	GGTGCAGGCATCCAGGTACT	CGGTGGGAAAGTGATGCTTAA
*HOXC*8	NM_022658.3	AACCCGTGCTCGCTTAGCT	GCCTCGTAGCCATAGAATTTGG
*HOXD3*	NM_006898.4	GGAGCTTCCTGAGTGCACAAT	CCTCCAAACAGTCCTGGGTTT
*HOXD8*	NM_019558.3	GGAATTTCTTTTTAACCCCTATCTGA	GCTAGGGCGTGGGAAACC
*FGFR1*	NM_023110.2	AACCTGACCACAGAATTGGAGGCT	ATGCTGCCGTACTCATTCTCCACA
*FGFR2*	NM_000141.4	TGCACAAGCTGACCAAACGT	CTGGACTCAGCCGAAACTGTT
*FGFR3*	NM_000142.4	GCGCCTGAGGCCTTGTTT	CCAAAGGACCAGACGTCACTCT
*FGFR4*	NM_002011.4	ACCCCACGCCCACCAT	TGCGGTTCTCCCCATGAA

*FGFR2, FGFR3 & FGFR4*

are incorrect. The table with the corrected gene sequences is included below.

